# Comparative Analysis of Fecal Microbiota in Vegetarians and Omnivores

**DOI:** 10.3390/nu15102358

**Published:** 2023-05-18

**Authors:** Changbao Sun, Ang Li, Cong Xu, Jiage Ma, Huan Wang, Zhanmei Jiang, Juncai Hou

**Affiliations:** 1 College of Food Science, Northeast Agricultural University, Harbin 150030, China; sunchangbao@126.com (C.S.); 15636116265@163.com (C.X.); jiage_ma@neau.edu.cn (J.M.); zhanmeijiang@neau.edu.cn (Z.J.); 2 College of Food and Biological Engineering, Qiqihar University, Qiqihar 161006, China; liang621liang@126.com; 3 Branch of Animal Husbandry and Veterinary of Heilongjiang Academy of Agricultural Sciences, Qiqihar 161005, China; whuan0517@126.com

**Keywords:** fecal microbiota, vegetarians, omnivores, obesity

## Abstract

Diet has a significant impact on fecal microbiota, which in turn plays an important role in human health. To evaluate the impact of dietary habits on fecal microbiota, we investigated the fecal microbial composition in vegetarians and omnivores using 16S rRNA gene sequencing, and estimated the correlation between fecal microbiota, body mass and diet. The dietary data showed that vegetarians consumed more plant-based foods rich in dietary fiber, omnivores consumed more animal-based foods rich in fat and overweight and obese people consumed more high-energy foods. Compared to omnivores, vegetarians had greater richness and diversity in their fecal microbiota. The *Firmicutes*/*Bacteroidetes* ratio was lower and the *Prevotella*/*Bacteroides* ratio was higher in vegetarians. The meat intake correlated positively with the proportion of *Bacteroides* and negatively with the proportion of *Prevotella*. The composition and diversity in fecal microbiota in the normal weight group, overweight group and obesity group were similar to that of vegetarians and omnivores, respectively. This paper revealed the distinctive characteristics of fecal microbiota in vegetarians and omnivores. The omnivorous diet contained more fat, which reduced the fecal microbial diversity, and was more likely to lead to being overweight or obese.

## 1. Introduction

The human intestine is inhabited by a great variety of microorganisms, which has profound effects on many aspects of human health such as the immune system [1], inflammatory disease [2] and obesity [3]. Increasing studies on the composition and diversity in fecal microbiota have been carried out in recent years [4,5,6,7]. Studies have shown that several factors such as host genotype, birth mode, age, use of antibiotics and dietary habits might alter the fecal microbial composition, and lead to chronic inflammation or metabolic dysfunction [8,9]. Additionally, the effects of the dietary factor on fecal microbial composition were dominant [10,11]. Long-term, relatively fixed dietary habits made the fecal microbial composition tend to be balanced and stable. It has been widely recognized that the vegetarian diet is a kind of healthy and therapeutic feeding type as it regulates the fecal microbial composition. In addition, it has been demonstrated that fecal microbiota regulate host energy metabolism and body mass [12]. Increasing evidence has supported the notion that vegetarians have a lower body mass index, lower serum cholesterol levels and lower prevalence rates of diabetes, cardiometabolic and other chronic diseases compared to omnivores [13,14]. Detailed comparative studies on fecal microbial composition among humans with different dietary habits are still scarce. To fill this knowledge gap, this study investigated the fecal microbial composition in vegetarians and omnivores and identified the distinctive characteristics of fecal microbiota that correlated with obesity.

## 2. Materials and Methods

### 2.1. Study Participants

In the present study, a total of 121 participants were recruited voluntarily via advertisement and telephone in the area of Harbin, China. All of the participants provided written informed consent before participating in this study. The participants were categorized as vegetarians (V, *n* = 46) and omnivores (R, *n* = 75). Vegetarians exclude meat and fish but may consume milk and eggs. Inclusion criteria: participants were required to be between the ages of 25 and 45, without chronic, infectious or intestinal diseases such as diabetes, irritable bowel syndrome, cancer and neurodegenerative disease; none had received any antibiotic treatment within at least six months prior to the study. Exclusion criteria: participants who are pregnant, breastfeeding, smoking or drinking. Vegetarians or omnivorous were those who had had this dietary lifestyle for at least one year before the study [15,16].

The trained interviewer collected information about the participants’ dietary and anthropometric data. The types of dietary habits were distinguished based on the consumption of food items in the last year. Quantitative and qualitative data on dietary intake were assessed using a semi-quantitative food frequency questionnaire (SQFFQ) as previously described in our works [17]. The energy and nutrient intake of each participant was calculated based on the Chinese food composition tables [18]. Participants’ height was measured without shoes within 0.1 cm using a research-grade digital stadiometer (HT-DM40, Faenza, Italy), and weight was measured without shoes in light clothing within 0.1 kg using an electronic scale (Yolanda-CS10A, Shenzhen, China). The nutritional status of all of the participants was checked based on their body mass index (BMI): underweight, BMI < 18.5; normal weight, 18.5 ≤ BMI < 24; overweight, 24 ≤ BMI < 28 or obese, BMI ≥ 28 [19]. BMI was calculated using the following formula: BMI = weight (kg)/height (m)^2^.

### 2.2. Fecal Sample Collection and DNA Extraction

The fecal samples of each participant were collected in sterile feces collection tubes with DNA stabilizer and stored at −80 °C. Microbial DNA was extracted from fecal samples using the E.Z.N.A. Stool DNA Kit (Omega Bio-tek, Norcross, GA, USA) according to the manufacturer’s protocols. The 16S rDNA V3-V4 region of the Eukaryotic ribosomal RNA gene was amplified via PCR (95 °C for 2 min, followed by 27 cycles at 98 °C for 10 s, 62 °C for 30 s and 68 °C for 30 s and a final extension at 68 °C for 10 min) using bacterial primers 341F: CCTACGGGNGGCWGCAG and 806R: GGACTACHVGGGTATCTAAT, where the barcodes are eight-base sequences unique to each sample [20]. PCR reactions were performed in triplicate. A 50.0 μL mixture containing 5.0 μL of 10 × KOD Buffer, 5.0 μL of 2.5 mM dNTPs, 1.5 μL of each primer (5.0 μM), 1.0 μL of KOD Polymerase and 100.0 ng of template DNA was used [21].

### 2.3. Analysis of 16S rRNA Sequences

Amplicons were extracted from 2% agarose gels, purified using the AxyPrep DNA Gel Extraction Kit (Axygen Biosciences, Union City, CA, USA) according to the manufacturer’s instructions and quantified using Quanti Fluor-ST (Promega, Madison, WI, USA), and purified amplicons were pooled in equimolar and paired-end sequenced (2 × 250) using an Illumina platform according to the standard protocols [16].

The raw data containing adapters or low-quality reads would affect the following reads’ assembly and analysis. Thus, to obtain high-quality clean reads, raw reads were further filtered according to the following rules: (1) removing reads containing more than 10% of unknown nucleotides (N); (2) removing reads containing less than 80% of bases with quality (Q-value) > 20. Paired-end clean reads were merged as raw tags using FLASH [22] (v 1.2.11) with a minimum overlap of 10 bp and mismatch error rates of 2%. Noisy sequences of raw tags were filtered using the QIIMF [23] (V1.9.1) pipeline under specific filtering conditions [24] to obtain high-quality clean tags. Clean tags were searched against the reference database [http://drive5.com/uchime/uchime_download.html (accessed on 12 August 2019)] to perform reference-based chimera checking using the UCHIME algorithm [http://www.drive5.com/usearch/manual/uchime_algo.html (accessed on 12 August 2019)]. All chimeric tags were removed and finally we obtained effective tags for further analysis. The effective tags were clustered into operational taxonomic units (OTUs) of ≥97% similarity using the UPARSE [25] pipeline.

### 2.4. Statistical Analysis

Statistical analysis was carried out using R [26] (version 3.5.0) software packages and in-house scripts. *p* < 0.05 was defined as a significant statistical difference. The age, height, weight and BMI data were represented as mean and standard deviation (mean ± SD).

The tag sequence with the highest abundance was selected as a reprehensive sequence within each cluster. Venn analysis between groups was performed in R to identify unique and common OTUs. The representative sequences were classified into organisms using a naive Bayesian model and an RDP classifier [27] (Version 2.2) based on the SILVA [28] database [https://www.arb-silva.de/ (accessed on 17 Aug 2019)]. The abundance statistics of each taxonomy and a phylogenetic tree were constructed in a Perl script and visualized using SVG. Chao1, Simpson and all other alpha diversity indexes were calculated in QIIME [29]. Statistical analyses of alpha diversity indexes between groups were calculated using Welch’s *t*-test and a Wilcoxon rank-sum test in R. Weighted and unweighted UniFrac distance matrixes were generated using QIIME. Multivariate statistical analyses, including principal component analysis (PCA), principal coordinates analysis (PCoA) and non-metric multidimensional scaling (NMDS) of (un)weighted UniFrac distances matrix, were conducted and plotted in R, as previously described [30].

## 3. Results

### 3.1. Characterization of Subjects

Twenty-one pieces of participants’ data were excluded from dietary analysis due to unqualified SQFFQ, and ultimately 100 participants (36 vegetarians and 64 omnivores) underwent dietary analysis and fecal microbiome analysis. According to the participants’ nutritional status, vegetarians were divided into a normal weight group (VN) and overweight group (VO); omnivores were divided into a normal weight group (RN), overweight group (RO) and obese group (RC).

Table 1 shows the characteristics of the 100 subjects. There were 48 males and 52 females. The average age, height, weight and BMI were 32.8 ± 4.8 years, 167.0 ± 7.4 cm, 65.1 ± 5.9 kg and 23.1 ± 3.1 kg/m^2^, respectively. The average BMI of omnivores was just numerically greater than that of the vegetarians. Similarly, the average age, height and weight were just numerically different between vegetarians and omnivores.

### 3.2. Dietary Profiles

**Figure 1:**
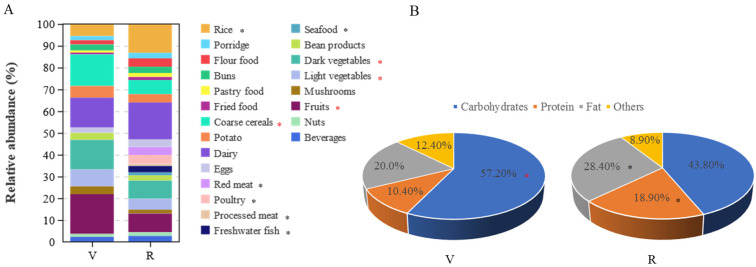
Food intake and macronutrient energy supply ratio of vegetarians (V) and omnivores (R). (**A**) Relative content of food intake in vegetarians and omnivores; (**B**) energy supply ratio of macronutrients in vegetarians and omnivores; red asterisks represent being significantly higher in vegetarians; black asterisks represent being significantly higher in omnivores.

The dietary habits and food intake of participants were surveyed using the SQFFQ. The survey results indicated that there was a significant difference in food intake between vegetarians and omnivores (Figure 1A). Compared to omnivores, vegetarians have significantly higher intakes of coarse cereals, vegetables and fruits, while they have significantly lower intakes of rice, meat and fish (*p* < 0.05).

The difference in macronutrient energy supply ratio between vegetarians and omnivores is shown in Figure 1B. The energy supply ratio of fat (28.4% vs. 20.0%) and protein (18.9% vs. 10.4%) in omnivores was significantly higher than that of vegetarians (*p* < 0.05), while the energy supply ratio of carbohydrates (43.8% vs. 57.2%) was significantly lower than that of vegetarians (*p* < 0.05). Compared with the dietary guidelines for Chinese residents [31], the energy supply ratios of carbohydrates, fat and protein in vegetarians were within the recommended range, while the energy supply ratios of fat and protein in omnivores were within the recommended range, and the proportion was large, but the energy supply ratio of carbohydrates was far lower than the recommended range.

### 3.3. Analysis of Fecal Microbial Composition in Vegetarians and Omnivores

**Figure 2:**
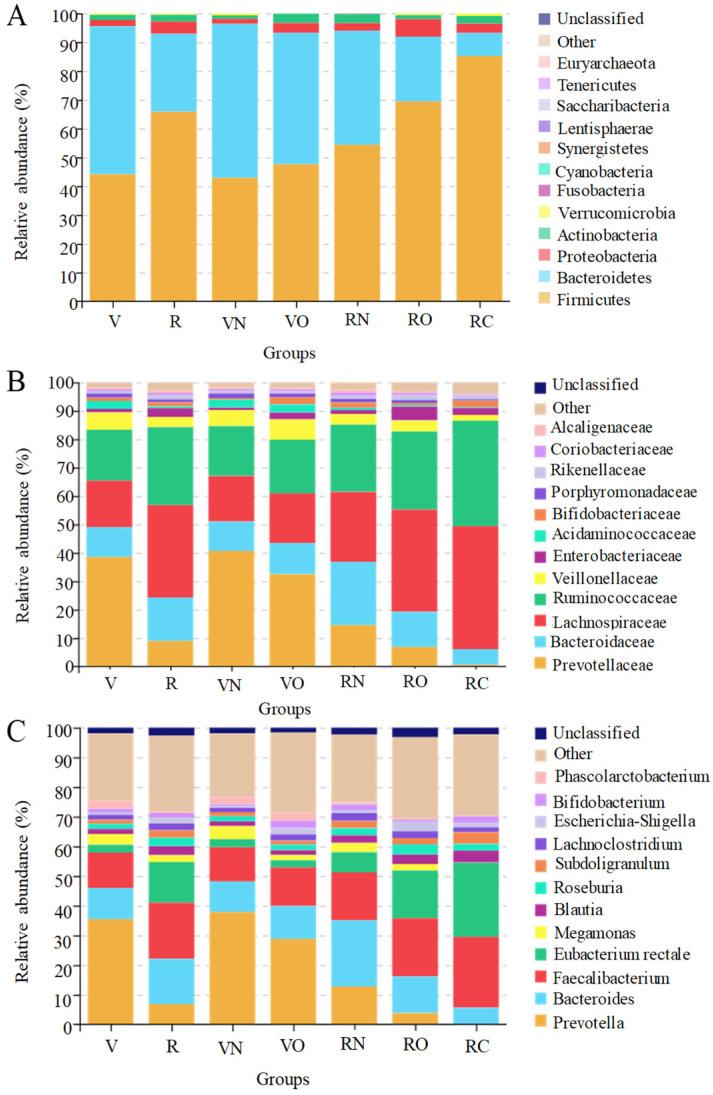
Composition of fecal microbiota in vegetarians and omnivores. (**A**) Phylum level; (**B**) family level; (**C**) genus level. V represents vegetarians; R represents omnivores; VN and VO represent normal weight group and overweight group for vegetarians, respectively; RN, RO and RC represent normal weight group, overweight group and obesity group in omnivores, respectively.

The gene sequencing results showed that a total of 7,824,057 reads were obtained from 100 fecal samples: 2,844,103 reads belonged to vegetarians with a mean value of 79,003 reads per sample, whereas 4,979,954 reads were from omnivores with a mean value of 77,812 reads per sample. From these reads, we identified an overall total of 769 OTUs, and of which 465 OTUs were common to all groups, while 179 and 125 OTUs were unique to vegetarians and omnivores, respectively. The identified OTUs were grouped into twelve phyla including *Firmicute*, *Bacteroidetes*, *Proteobacteria*, *Actinobacteria*, *Verrucomicrobia*, *Fusobacteria*, *Cyanobacteria*, *Synergistetes*, *Saccharibacteria*, *Euryarchaeota*, *Lentisphaerae* and *Tenericutes* (Figure 2A).

The relative abundance of *Firmicutes* and *Bacteroidetes* was the highest in the fecal samples of vegetarians and omnivores followed by *Proteobacteria* and *Actinobacteria* with >1% relative abundance, and the relative abundance sum of the four phyla accounted for more than 99.47% of the total bacteria. Each taxon was tested for differential abundance between vegetarians and omnivores. From the perspective of dietary habits, the abundance of *Actinobacteria*, *Firmicute* and *Proteobacteria* in vegetarians was significantly lower than that in omnivores (*p* < 0.05), and the abundance of *Bacteroides* was significantly higher than that in omnivores (*p* < 0.05). From the perspective of body mass index, in vegetarians, the abundance of *Firmicute*, *Proteobacteria* and *Actinobacteria* of the normal weight group (VN) was significantly lower than that of the overweight group (VO) (*p* < 0.05), while the abundance of *Bacteroides* and *Verrucomicrobia* in the normal weight group (VN) was significantly higher than that of the overweight group (VO) (*p* < 0.05); in omnivores, the abundance of *Firmicute*, *Proteobacteria*, *Actinobacteria* and *Verrucomicrobia* of the normal weight group (RN) was significantly lower than that of the overweight group (RO) and the obesity group (RC) (*p* < 0.05), while the abundance of *Bacteroides* and *Fusobacteria* in the normal weight group (RN) was significantly higher than that of the overweight group (RO) and obesity group (RC) (*p* < 0.05). The abundance of *Firmicute* increased with increasing weight, while the abundance of *Bacteroides* decreased with increasing weight, leading to an increased *Firmicutes*/*Bacteroides* ratio with increased weight. The *Firmicutes*/*Bacteroides* ratio (0.86 vs. 2.43) in the vegetarians was smaller than that in the omnivores, and the results indicated a significant difference in the fecal microbial composition between vegetarians and omnivores.

A total of 46 families of bacteria were detected in the fecal samples of vegetarians and omnivores. Of these families, those with relative abundance greater than 1% in all of the groups included *Ruminococcaceae*, *Lachnospiraceae*, *Bacteroidaceae*, *Veillonellaceae*, *Acidaminococcaceae*, *Porphyromonadaceae*, *Enterobacteriaceae*, *Rikenellaceae*, *Bifidobacteriaceae*, *Prevotellaceae*, *Coriobacteriaceae* and *Alcaligenaceae* (Figure 2B). The sum of the relative abundance of *Prevotellaceae*, *Bacteroidaceae*, *Lachnospiraceae*, *Ruminococcaceae*, *Veillonellaceae* and *Enterobacteriaceae* accounts for more than 90.65% of the total bacteria; these form the most dominant bacteria family in vegetarians and omnivores.

At the family level, there was more discrepancy in fecal microbial composition between vegetarians and omnivores. The abundance of *Prevotellaceae*, *Veillonellaceae* and *Acidaminococcaceae* in vegetarians was significantly higher compared with omnivores (*p* < 0.05), while the abundance of *Bacteroidaceae*, *Ruminococcaceae*, *Lachnospiraceae*, *Enterobacteriaceae* and *Rikenellaceae* was significantly lower compared with omnivores (*p* < 0.05).

From the perspective of body mass index, in vegetarians, the abundance of *Prevotellaceae*, *Porphyromonadaceae* and *Rikenellaceae* in the normal weight group (VN) was significantly higher compared with the overweight group (VO) (*p* < 0.05), and the abundance of *Ruminococcaceae*, *Lachnospiraceae* and *Veillonellaceae* in the normal weight group (VN) was significantly lower compared with the overweight group (VO) (*p* < 0.05); in omnivores, the abundance of *Prevotellaceae*, *Bacteroidaceae*, *Veillonellaceae*, *Acidaminococcaceae*, *Porphyromonadaceae*, *Coriobacteriaceae* and *Veillonellaceae* in the normal weight group (RN) was significantly higher compared with the overweight group (RO) and obesity group (RC) (*p* < 0.05), whilst the abundance of *Ruminococcaceae*, *Lachnospiraceae*, *Enterobacteriaceae* and *Rikenellaceae* in the normal weight group (RN) was significantly lower compared with the overweight group (RO) and obesity group (RC) (*p* < 0.05).

The genera with a high abundance (>1%) detected in all of the groups were shown in Figure 2C, including Prevotella, Faecalibacterium, Bacteroides, Eubacterium rectale, Megamonas, Blautia, Roseburia, Subdoligranulum, Lachnoclostridium, Escherichia-Shigella, Bifidobacterium and Phascolarctobacterium. Those with the highest abundance of 35.46% and 18.91% found in vegetarians and omnivores were Prevotella and Faecalibacterium, respectively. The abundance of Prevotella, Megamonas and Phascolarctobacterium in vegetarians was significantly higher than that in omnivores (*p* < 0.05), whereas the abundance of Faecalibacterium, Lachnoclostridium, Bacteroides, Eubacterium rectale, Blautia, Roseburia, Subdoligranulum and Escherichia-Shigella in vegetarians was significantly lower than that of omnivores (*p* < 0.05).

From the perspective of body mass index, in vegetarians, the abundance of *Prevotella* and *Megamonas* in the normal weight group (VN) was significantly higher than that of the overweight group (VO) (*p* < 0.05), and the abundance of *Faecalibacterium* and *Lachnoclostridium* in the normal weight group (VN) was significantly lower than that of the overweight group (VO) (*p* < 0.05); in omnivores, the abundance of *Prevotella*, *Megamonas*, *Bacteroides* and *Phascolarctobacterium* in the normal weight group (RN) was significantly higher compared with the overweight group (RO) and obesity group (RC) (*p* < 0.05), whilst the abundance of *Faecalibacterium*, *Eubacterium rectale*, *Blautia* and *Roseburia* in the normal weight group (RN) was significantly lower compared with the overweight group (RO) and obesity group (RC) (*p* < 0.05).

### 3.4. Analysis of Fecal Microbial Diversity in Vegetarians and Omnivores

To evaluate the overall differences in fecal microbial composition between vegetarians and omnivores, we calculated the alpha diversity indexes (Chao1, Ace, Shannon and Simpson). Compared to omnivores, the higher alpha diversity indexes were obtained for vegetarians (Table 2), and with an increase in BMI, the alpha diversity indexes decreased in vegetarians and omnivores, indicating that the alpha diversity in vegetarians was higher than that in omnivores and that the alpha diversity in the normal weight groups was higher compared with the overweight groups and obesity group.

**Figure 3:**
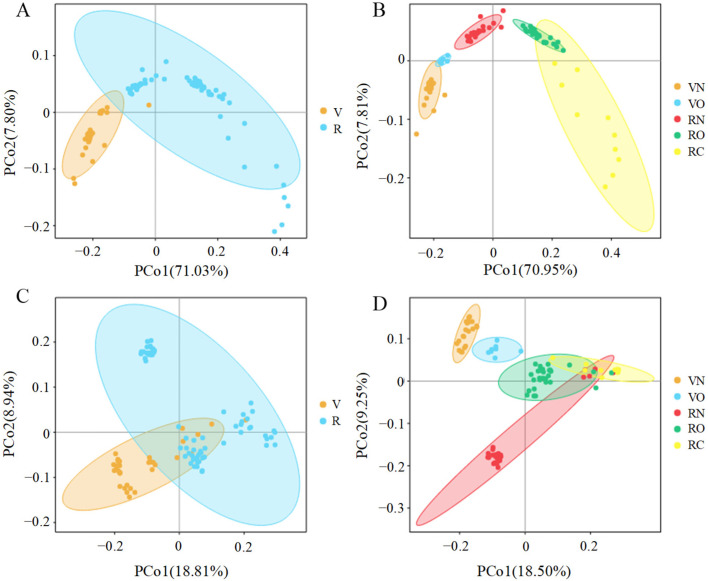
Principal coordinates analysis (PCoA) plots of fecal microbiota in vegetarians (V) and omnivores (O). The plots show the first two principal coordinates axes using a weighted (**A**,**B**) and unweighted (**C**,**D**) UniFrac distance matrix. VN and VO represent the normal weight group and overweight group in vegetarians, respectively; RN, RO and RC represent the normal weight group, overweight group and obesity group in omnivores, respectively.

To show the differences between microbial community structures in vegetarians and omnivores, we calculated the beta diversity parameter, including the unweighted and weighted UniFrac distance matrix (PCA, PCoA and NMDS). Due to the similar trend of all of the analysis results, only the results of the PCoA are shown in Figure 3. Figure 3A,B shows that the first coordinate (PCo1) explains 71.03% of the inter-sample variance (*p* < 0.05), while Figure 3C,D explains the 18.81% in vegetarians versus omnivores (*p* < 0.05). In subgroups based on BMI, we found that the samples in the same groups were gathered, the samples among groups were well differentiated and that there was a clear boundary between two vegetarian groups (VN and VO) and three omnivorous groups (RN, RO and RC) (*p* = 0.01; Figure 3B,D).

### 3.5. Correlation between Food and the Fecal Microbial Community

**Figure 4:**
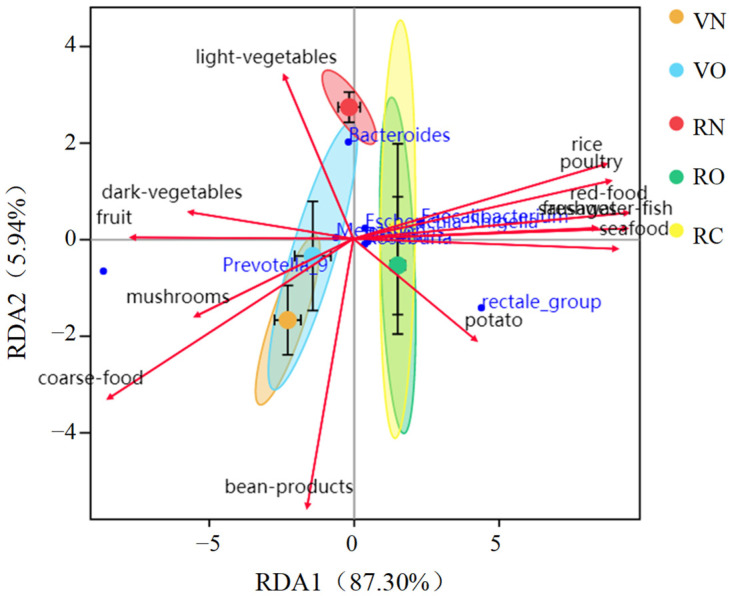
Redundancy analysis (RDA) of the correlation between food intake and fecal microbiota. VN and VO represent the normal weight group and overweight group in vegetarians, respectively; RN, RO and RC represent the normal weight group, overweight group and obesity group in omnivores, respectively.

To investigate the correlation between food intake and fecal microbiota, we conducted a redundancy analysis (RDA) on vegetarians and omnivores. Variation in fecal microbiota correlated significantly with individual food intake variation (Figure 4). The result showed that omnivores consumed more rice and animal-based foods, and vegetarians consumed more coarse food and plant-based foods. Compared to the normal weight group, daily fat intake was significantly higher in the overweight group and obesity group, the intake of plant-based foods rich in dietary fiber correlated positively with the genera *Prevotella*, *Megamonas* and *Phascolarctobacterium* and the intake of animal-based foods rich in fat and protein correlated positively with the genera *Faecalibacterium*, *Eubacterium rectale*, *Blautia* and *Escherichia-Shigella*. The correlation between altered fecal microbiota and high BMI suggested that high-fat-diet-associated obesity was present among the omnivores.

## 4. Discussion

The interaction between diet and fecal microbiota has been increasingly studied [32,33]. Thus, the beneficial use of diet to regulate the fecal microbial composition to improve human health has proven to be an effective nutritional treatment method [34]. Here, we investigated the distinctive influence of vegetarian diet and omnivorous diet on the fecal microbial composition.

The diversity in fecal microbiota reflected the stability of the microecology and the ability to resist the invasion of external pathogenic bacteria. Low diversity did not necessarily mean illness, but it meant one was more susceptible to factors such as diet, environment or disease. Higher diversity often led to healthier physical conditions [35]. A significant difference in the alpha diversity index between vegetarians and omnivores was found, with vegetarians displaying greater richness. This was related to the fact that vegetarians mainly consumed plant-based foods. A plant-based diet contains rich dietary fiber, which is the main nutrient source for fecal microbiota. The number of fecal microbiota increased due to the increase in nutrients, which in turn increased the abundance of fecal microbiota [35]. Additionally, richer fecal microbiota is advantageous to the host since greater taxonomic richness might also mean greater functional diversity. The current study found that the alpha diversity in the fecal microbiota in the normal weight group was higher compared with the overweight group and obesity group. The present results correspond to the proposals of Chen et al. [36] and Liu et al. [37]. The beta diversity in the fecal microbiota reflected the microbial overall composition and richness. The more similar the dietary structure of the study subjects, the more similar the composition of the fecal microbiota, and the closer the spatial distance of the samples. Thus, in the present research, the dietary habits of vegetarians and omnivores were different, leading to a significant difference in the fecal microbial composition. These results are congruent with the study of De Filippo et al. [38].

Vegetarians were reported to have lower frequencies of obesity, hypertension, diabetes and cardiovascular disease [39,40]. Vegetarians consumed more plant-based foods that were rich in dietary fiber and micronutrients, such as coarse grains, vegetables and fruits, while they ate fewer animal-based foods rich in fat such as meat, poultry and fish. Increasing evidence has shown that a vegetarian diet is beneficial to reduce oxidative stress. Additionally, a large amount of dietary fiber could stimulate fecal microbiota metabolism to produce more short-chain fatty acids (SCFAs), mainly acetate, propionate and butyrate. Diet provided a variety of nutrients and energy for the growth and reproduction of fecal microbiota. In turn, this caused differences in the fecal microbial composition. This study found that the fecal microbial composition of vegetarians was more favorable compared with omnivores with fewer *Firmicutes* and more *Bacteroidetes*, similar to the results of Wu et al. [41]. Up-to-date knowledge has suggested that a high abundance of *Firmicutes* leads to an imbalance of fecal microbial composition, which can cause metabolic dysfunction and induce obesity, hypertension, diabetes and other metabolic diseases [42]. The current research found a higher abundance of *Firmicutes* and a lower abundance of *Bacteroidetes* in omnivores compared with vegetarians, which might be attributed to the differences in body mass index. Evidence from animal and human studies has demonstrated that obesity is related to an increased *Firmicutes*/*Bacteroidetes* ratio [43,44]. A study reported by Ley et al. [45] showed that a lower *Firmicutes*/*Bacteroidetes* ratio was associated with the lean phenotype, which was generally considered to be beneficial for health. It was suggested that the content and proportion of *Firmicutes* and *Bacteroidetes* could be used as characteristics to distinguish between the microbial communities in lean and obese people [46]. *Firmicutes* and *Bacteroidetes* were the two more abundant bacteria in the intestine. *Bacteroides* has a high degree of functional redundancy, while *Firmicutes* was composed of core bacteria with multiple metabolic functions. The fecal microbiota in obesity could increase energy gain from food [47]. The metabolite of *Bacteroides* was mainly propionate, which was absorbed by the colon and transported to the liver as the substrate of gluconeogenesis to maintain energy balance, inhibited the synthesis of fat and cholesterol and had a lipid-lowering effect. Analyzing particularly the *Firmicutes* subpopulations, the results of the present study found an increase in the abundance of two acetate-producing bacteria—*Faecalibacterium* and *Eubacterium rectale* in omnivores and the obesity group in comparison with vegetarians and the normal weight group. Acetate was a major energy source for the body cells and stimulated the expression of adipocyte differentiation factors, contributing to adipocyte proliferation and fat deposition [48]. The abundance of *Bacteroidetes* mainly composed of the genera *Prevotella* and *Bacteroides* which were higher in vegetarians than in omnivores. Both *Prevotella* and *Bacteroides* were commonly presented in the human intestine. Our study observed that there was a higher *Prevotella*/*Bacteroides* ratio in vegetarians and the normal weight group than in omnivores and the obesity group. Likewise, several studies have suggested that there is a higher abundance of *Prevotella* in individuals who consume plant-based food and a predominance of *Bacteroides* in individuals who consume animal-based food [49,50]. This result might be due to colonic fermentation, which could inhibit some fecal microbiota. The reason for this might be that dietary fiber from plant-based food is fermented by fecal microbiota to produce more short-chain fatty acids, which in turn leads to a decrease in pH from 6.5 to 5.5. *Bacteroides* did not grow well under pH 5.5 conditions [51]. This might also be the reason why there was a lower abundance of *Bacteroides* in vegetarians and the normal weight group than in omnivores and the obesity group. Other studies have shown that legumes in a vegetarian diet can increase the proportion of *Megamonas*, *Bifidobacterium* and *Phascolarctobacterium*, but reduce the proportion of *Bacteroides* [52]. *Phascolarctobacterium* focus on utilizing succinate salts produced by *Bacteroides*, and the proportion of *Bacteroides* increased due to a high-fat diet and was positively correlated with body weight. Normal-weight individuals had a higher abundance of *Phascolarctobacterium* in their intestines, making it an indicator for predicting obesity [53]. Species related to obesity, such as *Faecalibacterium*, *Bacteroides*, *Eubacterium rectale*, *Brucella*, *Roseburia* and *Escherichia-Shigella*, have been increasingly confirmed in research. The ability of *Faecalibacterium* to utilize carbohydrates from dietary sources was quite limited, and it could not grow on starch or hemicellulose, while a high saturated fat diet could increase the proportion of *Faecalibacterium*. *Brucella* and *Roseburia* have an extraordinary ability to utilize carbon dioxide and hydrogen or formic acid to produce acetate, which is related to adipocyte differentiation and fat deposition. The diversity in fecal microbiota in obese individuals decreased, intestinal integrity was disrupted, intestinal permeability was enhanced, harmful substances such as endotoxins were absorbed more and the body’s ability to obtain energy from the diet was increased.

In this study, we found a significant difference in the fecal microbial composition between vegetarians and omnivores and a correlation between obesity and fecal microbiota. This study has limitations due to a relatively small sample size and a lack of in-depth biological and biochemical information, not allowing it to address the complex physiological link between diet, fecal microbiota and phenotypes.

## 5. Conclusions

The current research revealed the distinctive characteristics in fecal microbial diversity and composition in vegetarians and omnivores. A vegetarian diet with high fiber might increase fecal microbial diversity. An omnivorous diet containing more fat and calories might reduce fecal microbial diversity, and is likely to lead to being overweight or obese. This study provides a new theoretical basis for future research into a dietary intervention to regulate the balance of fecal microbial composition and the obesity phenotypes, and the development of precision nutrition.

## Figures and Tables

**Table 1:** Characterization of subjects.

Characteristics	Total (*n* = 100)	Vegetarians (*n* = 36)	Omnivores (*n* = 64)	*p*
Age (years), mean ± SD	32.8 ± 4.8	33.6 ± 5.1	32.3 ± 4.6	0.091
Height (cm), mean ± SD	167.0 ± 7.4	165.0 ± 7.3	168.0 ± 7.2	0.086
Weight (kg), mean ± SD	65.1 ± 5.9	59.5 ± 4.9	68.1 ± 6.3	0.051
BMI (kg/m^2^), mean ± SD	23.1 ± 3.1	21.8 ± 2.5	23.8 ± 3.2	0.063
Male/Female	48/52	16/20	32/32	-

**Table 2:** Comparative analysis of alpha diversity index for each group.

Groups	Chao1	Ace	Shannon	Simpson
V	964.69 ± 134.63 *	947.53 ± 98.65 *	5.58 ± 0.01 *	0.93 ± 0.01
R	899.03 ± 151.82	880.33 ± 152.42	5.46 ± 0.58	0.91 ± 0.04
VN	976.22 ± 235.67 ^a^	948.27 ± 227.49 ^a^	5.66 ± 0.72 ^a^	0.94 ± 0.05 ^a^
VO	959.53 ± 134.63 ^b^	947.20 ± 98.65 ^a^	5.39 ± 0.01 ^bc^	0.92 ± 0.01 ^a^
RN	949.34 ± 134.95 ^bc^	923.77 ± 130.16 ^a^	5.85 ± 0.51 ^a^	0.95 ± 0.03 ^a^
RO	886.36 ± 174.57 ^d^	869.55 ± 180.84 ^b^	5.40 ± 0.63 ^b^	0.91 ± 0.05 ^a^
RC	810.23 ± 40.35 ^e^	802.99 ± 44.57 ^c^	5.31 ± 0.04 ^bd^	0.89 ± 0.01 ^a^

Note: V represents vegetarians; R represents omnivores; VN and VO represent normal weight group and overweight group in vegetarians, respectively; RN, RO and RC represent normal weight group, overweight group and obesity group in omnivores, respectively; the asterisks indicate that the difference was significant between vegetarians and omnivores (*p* < 0.05); different letters in the same column indicate that there was a significant difference between groups (*p* < 0.05).

## Data Availability

The data presented in this study are available on request from the corresponding author. The data are not publicly available due to privacy.
